# A Simple, Dynamic Geometric Phantom for MRI and CT Reconstruction Pipelines: Beyond Shepp–Logan

**DOI:** 10.1002/mrm.70495

**Published:** 2026-07-03

**Authors:** Tamás Hakkel, Noémi Kovács, József Sinkó, Domokos Máthé, Krisztián Szigeti

**Affiliations:** ^1^ Department of Biophysics and Radiation Biology Semmelweis University Budapest Hungary; ^2^ Mediso Medical Imaging Systems Ltd Budapest Hungary; ^3^ Hungarian Centre of Excellence for Molecular Medicine (HCEMM) Szeged Hungary

**Keywords:** constructive solid geometry, CT, dynamic phantom, Julia, MRI, Shepp–Logan

## Abstract

A customizable, dynamic geometric phantom was developed to bridge the gap between simplistic static phantoms and highly realistic, complex 4D solutions. The phantom enables initial testing, helps detect implementation errors, and supports quantitative evaluation of new dynamic acquisition and reconstruction methods. It is formed from a series of superellipsoids using the constructive solid geometry technique and serves as a schematic representation of a human torso, including the lungs, liver, and stomach, as well as a four‐chamber heart, major vessels, and simplified skeletal structures. This design allows full parameterization of voxel intensities and organ volumes. Respiratory and cardiac motions are modeled using built‐in, decoupled signal generators. A high‐performance, open‐source reference implementation was developed in Julia and is available both as a library and a standalone console application. The phantom's capabilities were demonstrated through four experiments. The difference between the input and measured lung volumes was quantified, yielding a maximum relative error of 5.5% and a median error of 2.5%. The relative error of the cardiac chamber volumes ranged from 1.76% to 3.67%. Performance was benchmarked across multiple phantom sizes and thread counts, and a realistic Cartesian MRI simulation demonstrated how the phantom can be used to quantify motion blur and artifacts. The proposed dynamic geometric phantom provides an intermediate option between simple static phantoms and complex, proprietary, anatomically realistic 4D models. It provides a highly customizable, easy‐to‐use, open‐source tool for researchers developing new reconstruction algorithms, and it can be easily integrated into existing reconstruction pipelines.

## 1 Introduction

Medical imaging has undergone rapid evolution since its inception, and new imaging methods continue to appear in the literature. During the development of a new acquisition or reconstruction method, simulation plays a crucial role: It enables early testing to reveal implementation errors and provides a ground‐truth dataset for quantitative evaluation. Although error quantification is often possible—and in some cases more accurate—using repeated high‐resolution in vivo or ex vivo measurements, it becomes extremely challenging in dynamic imaging, especially in dynamic MRI, where high spatial resolution inherently limits temporal resolution, and vice versa. Simulation is therefore essential for developing methods in dynamic imaging.

As a result, the current landscape of in silico and in vitro phantoms is diverse, offering specialized solutions for a wide range of use cases. One of the earliest digital phantoms is the Shepp–Logan phantom, a simple geometric 2D phantom consisting of ten overlapping ellipses [1]. Despite its highly simplified depiction of the human head, its simplicity made it popular, and it is still frequently used for CT and MRI simulations. Several 3D extensions of the phantom have been proposed. One of the original authors, L. A. Shepp, proposed a 17‐ellipsoid model for a more realistic representation [2], but it never became as widely used as the 2D version. Currently, two other 3D extensions are commonly used: (i) the model designed by A. C. Kak and M. Slaney in 1988 [3], which uses the same number of ellipsoids as the 2D version in a slightly different arrangement, and (ii) the model proposed by C. Jacobson in her PhD thesis in 1996 [4], which matches the original 2D phantom at axial slice z=0.25. Implementing these phantoms is easy, and multiple open‐source implementations are available. Another advantage is that sampling can be performed at arbitrary grid densities.

For greater anatomical realism, MRI researchers often use the Brainweb dataset [5], a digital phantom derived from real MRI acquisitions that models ten brain tissue types. This phantom consists of a discrete 3D tissue map and requires interpolation when a sampling grid other than the original is required. For CT, numerous specialized phantoms are available, including hundreds of dosimetry phantoms [6], the FORBILD [7] and the Zubal voxel phantoms [8] for validating reconstruction methods, and the virtual imaging clinical trial for regulatory evaluation (VICTRE) for clinical evaluation [9]. In addition, many studies discuss the design of in vitro phantoms [10, 11].

In contrast, 4D phantoms suitable for simulating dynamic imaging are available in much smaller numbers. The most widely used options are the phantoms developed by researchers at Duke University and Johns Hopkins Medical Institutions, who created multiple generations of CT‐derived phantoms that simulate respiratory and cardiac motion with high realism. Their latest phantoms—4D XCAT, MOBY, and ROBY—model the full anatomy of the human, mouse, and rat, respectively [12, 13]. These phantoms are built from non‐uniform rational B‐splines (NURBS) and subdivision surfaces, which allow the definition of complex anatomical structures and voxelization on an arbitrarily dense grid. Their availability, however, is restricted because they require a commercial license. More application‐specific phantoms include the fetal brain magnetic resonance acquisition numerical phantom (FaBiAN) [14], which simulates stochastic fetal motion, and the abdominal MR phantom with respiratory motion of the abdominal organs [15, 16], both available as open‐source software. Additionally, in vitro phantoms have been proposed to simulate respiratory and cardiac motion [14, 15].

The phantom presented in this work aims to fill the gap between simple, publicly available static phantoms, such as Shepp–Logan, and sophisticated, anatomically realistic 4D torso phantoms, such as XCAT. The proposed torso phantom is constructed from simple geometric shapes, enabling high‐speed rendering. It resembles a human torso for visual inspection but does not aim for anatomical accuracy. Instead, it provides full control over tissue intensities and motion parameters via input arguments: The intensity of each represented organ can be easily adjusted, and lung and cardiac volumes over time can be configured. Additionally, it features a high‐performance, open‐source implementation released under a permissive MIT license.

## 2 Methods

### 2.1 Geometric Primitives

To allow rendering on arbitrarily dense grids, an analytical definition of the phantom is required. While splines are commonly used to describe complex shapes, constructive solid geometry provides a more effective way to construct schematic representations of real‐life objects. Constructive solid geometry defines simple shapes, called geometric primitives, and combines them using Boolean operations, such as union, difference, and intersection. The main advantage of this technique is that it requires only a small number of parameters and allows efficient implementation. Because most organs in the human body have curved shapes, ellipsoids are natural candidates as geometric building blocks. However, superellipsoids—generalizations of ellipsoids—offer greater flexibility for modeling schematic organs, as they can represent both square and conical shapes. A superellipsoid is defined as the set of points satisfying the inequality:

(1) $$\left({\left|\frac{x}{a}\right|}^{r}+{\left|\frac{y}{b}\right|}^{s}+{\left|\frac{z}{c}\right|}^{t}\right)\leq 1$$

where (x,y,z) denote the coordinates of a point, while a, b, and c are the semi‐axes of the shape, and the exponents r, s, and t control the roundness and sharpness of the shape. If an exponent is higher than two, the shape becomes more square‐like in the corresponding direction, and lower exponents promote conical corners.

### 2.2 Anatomical Construction

Although the phantom is not anatomically realistic, its schematic representation of the major organs facilitates visual evaluation of the reconstructed image. The phantom includes representation of the lungs, a four‐chamber heart, the aorta, the pulmonary artery, the superior vena cava, the spine, the ribs, the arms, the stomach, and the liver. For simplicity, the torso base is represented as a homogeneous tissue. The intensity values of all organs are parametrized and can be easily adjusted. The lungs are modeled as the union of two superellipsoids that are more conical superiorly and more square‐like inferiorly. Two additional superellipsoids are subtracted from the inferior part of each lobe to represent the diaphragm. Also, another superellipsoid, referred to as pericardum, is subtracted from the center of the lungs for technical reasons, to eliminate the dependence between lung and heart volumes.

The heart is modeled in greater detail, including the myocardium, left and right ventricles, and both atria. The heart is asymmetric in our representation, with the left ventricle and atrium larger than their right‐sided counterparts. For simplicity, the heart is modeled without rotation and is aligned with the *z*‐axis. The vessels are modeled as slightly curved, yet geometrically simple structures.

The skeletal structures include the spine, modeled as a series of vertebra‐like elements with a curvature mimicking the natural curvature of the human spine. The phantom also includes ribs modeled on a tilted plane, with the anterior portions lying inferior to the posterior segments where they attach to the spine. The ribs also differ in how much the full circumference of the torso is covered: The upper ribs go all around a full ellipsoid, but the lower ones are open toward the anterior side of the body. The arms also contain simplified bone‐like structures connected to the spine through minimalistic representations of the scapulae. The liver and the stomach are also modeled using superellipsoids. The superellipsoids representing the liver are on the right side, slightly inferior to the diaphragm, and the stomach resides on the left side, also beneath the diaphragm.

For the exact calculation of the positions and radii of the superellipsoids, refer to the corresponding documentation page: https://hakkelt.github.io/GeometricMedicalPhantoms.jl/v1.0.3/phantoms/phantom_specification/.

### 2.3 Modeling Dynamics

The core novelty of the phantom is that the dynamics of organ volume changes over time are fully parametrizable. Although anatomical accuracy was not the goal, the modeled respiratory and cardiac motions mimic key aspects of human physiology. In essence, respiratory motion modulates lung and chest volumes and induces positional changes in the diaphragm, ribs, heart, liver, and stomach. The diaphragm, liver, and stomach move predominantly in an inferior‐superior direction during respiration, and all internal organs exhibit anterior–posterior displacement at different amplitudes, reflecting their non‐stationary positions in humans. To reduce model complexity, cardiac motion is decoupled from the motion of other organs: The pericardial radius is defined by the maximal heart size, and it remains constant throughout the time frames; otherwise, the lung volume would also be affected by the cardiac cycle.

**FIGURE 1:**
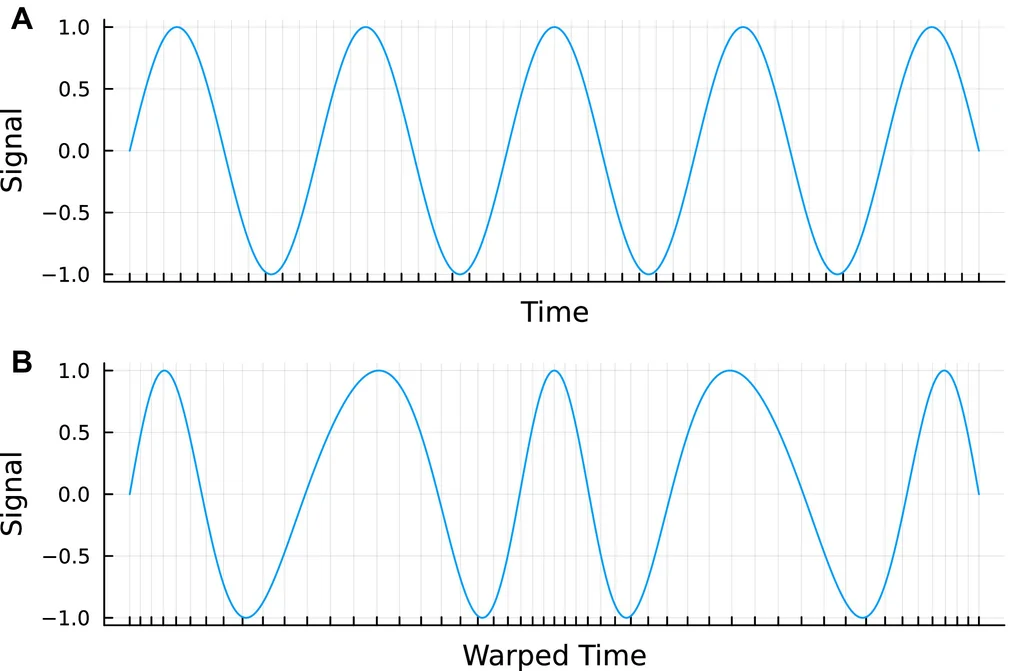
Visualization of the sinusoidal time warping used by signal generators to simulate natural variability of the rate of physiological signals over time. Subplot A shows the original sinusoidal signal (an ordinary sine wave in this case) on a uniform time grid, and subplot B shows the same signal after time warping. The time warping is implemented as a sinusoidal perturbation of the phase trajectory, which results in a non‐uniform stretching and compression of the signal over time, mimicking natural variability in physiological signals. The ticks on the *x*‐axis of subplot B indicate the original time points, which are no longer uniformly spaced after warping.

To facilitate practical use, the phantom's definition includes default respiratory and cardiac signal generators that reproduce several characteristic features of real physiological signals. The respiratory signal is generated as a compound sinusoid, mapped to physiologically plausible lung‐volume limits. Generation of the signal involves several steps: First, the program defines a phase trajectory based on the breathing frequency, with an optional low‐frequency perturbation enabled by default. This perturbation is implemented as a sinusoidal time warping, visualized by Figure 1. Next, an asymmetric base waveform is constructed as the sum of two sinusoidal components, the second term being the harmonic of the first. Finally, the waveform is modulated by an amplitude‐modulation component that simulates baseline wander.

The cardiac signal generator can generate realistic chamber volumes. These volumes are synthesized on a discrete time grid using a periodic phase signal derived from the heart rate, incorporating a mild time‐warp to simulate natural heart rate variability and avoid perfectly periodic cycles. Using that phase signal, the program partitions each cardiac cycle into time‐varying systolic fractions. Chamber volumes are defined piecewise over systole and diastole. Ventricular volumes follow a cubic‐like systolic ejection and nonlinear diastolic refilling, and a term for a late‐diastolic Gaussian atrial‐kick. Atria volumes exhibit systolic filling and diastolic emptying with an additional Gaussian contraction notch. Finally, slow multiplicative amplitude modulation and additive baseline wander are applied to introduce physiologically plausible long‐term variability.

### 2.4 Reference Implementation

Despite the phantom's relative simplicity compared with more realistic alternatives, implementing it is a nontrivial task, especially when performance is a concern. We therefore provide a reference implementation that is easy to use while offering high‐speed rendering. We implemented this rendering software in Julia [16]. We chose this language because it offers high execution speed characteristics, maintains excellent readability, and provides native, efficient support for multidimensional arrays and complex mathematical operations.

The software package consists of three main logical units. The primitives layer implements fundamental geometric building blocks—such as ellipsoids and superellipsoids—together with optimized rendering functions that efficiently map these analytic shapes onto discrete grids. Building these primitives, the phantom‐definition layer provides functions to parametrize, construct, and render the phantom. The highest level of the software provides a console application with powerful export capabilities to numerous formats, including npy (array file format for Python's NumPy library), mat (MATLAB's array file), cfl/hdr (custom format for the BART toolbox [17]), NIfTI, png, multi‐page tiff, and animated gif.

The software is distributed in two primary formats: a programming library and a standalone executable. The core functions are registered as the Julia package GeometricMedicalPhantoms.jl, which is also accessible via a C‐callable binary and dedicated Python and MATLAB wrappers. Additionally, we provide a pre‐compiled, command‐line application that bundles all dependencies, allowing users to run the software immediately after extraction without a formal installation process. This standalone application is available on all major desktop platforms (Windows, Linux, and macOS) and across multiple processor architectures (x86‐64 on all operating systems, and ARM on macOS). In addition to the torso phantom described in this article, the software package also includes a 3D Shepp–Logan phantom and a “tubes” phantom for testing multi‐echo imaging pipelines.

**FIGURE 2:**
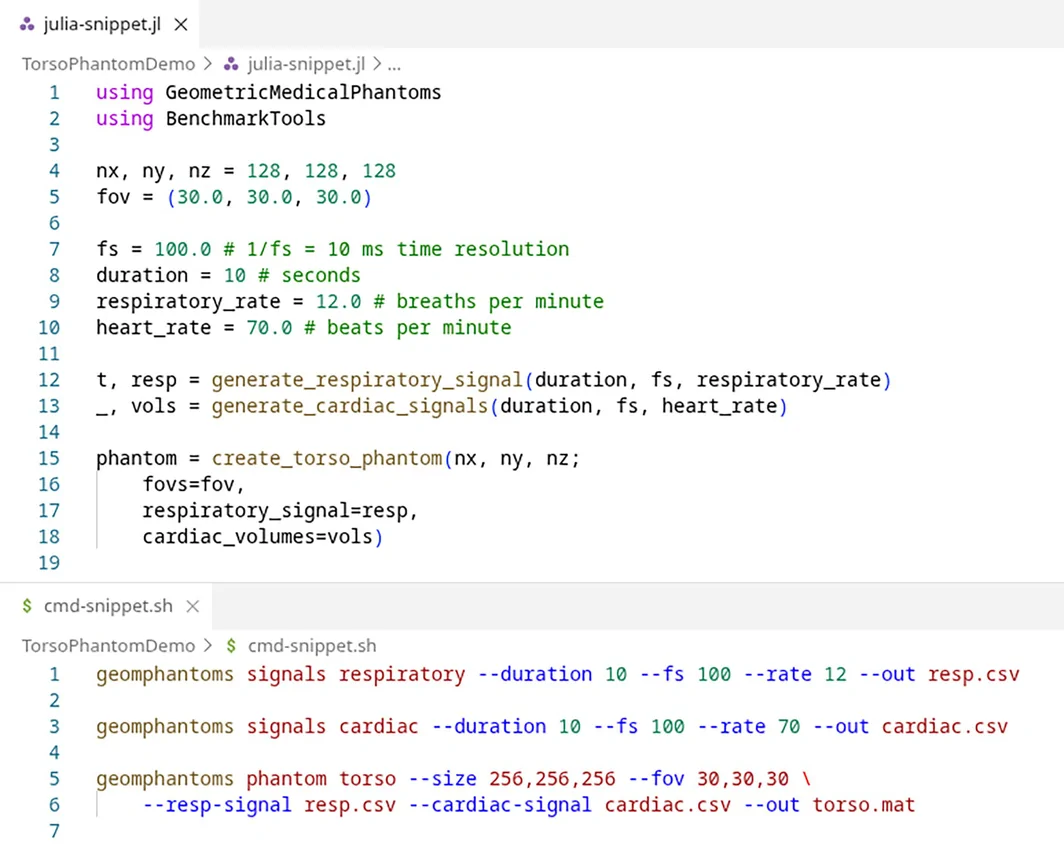
Julia code snippet (top) and a bash script (bottom) that render the 4D phantom using the library and the command‐line interface of the reference implementation.

Figure 2 shows a Julia code snippet that renders the dynamic phantom using the built‐in respiratory and cardiac signal generators, together with the equivalent console commands for generating the same dataset via the command‐line interface.

## 3 Results

In this section, we demonstrate the features and capabilities of the phantom definition and the reference implementation through four experiments.

**FIGURE 3:**
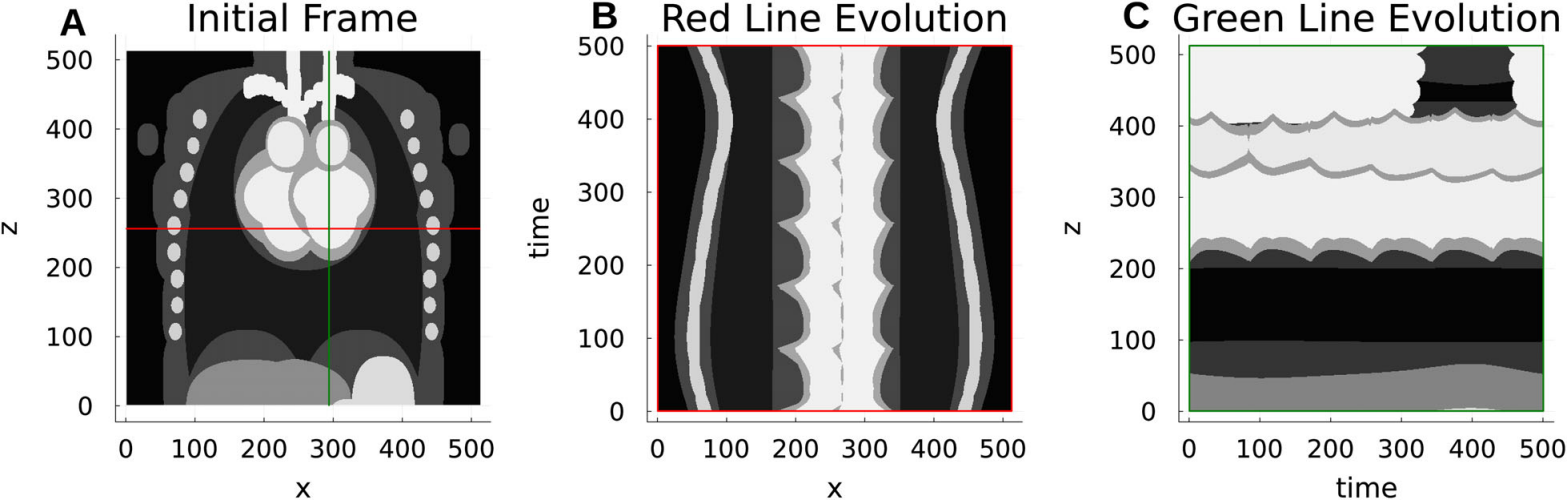
The central coronal slice of a single frame (A), change of a single horizontal line along time (B), and change of a single vertical line along time (C).

Experiment A illustrates the cardiac and respiratory dynamics by plotting the changes in values along two lines. Figure 3 displays a single frame and the changes in values along a horizontal and a vertical line over time.

Experiment B quantifies the difference between the input volumes and the volumes computed from voxel count for the lung and the four cardiac chambers. The reason behind this experiment is that exact volume control is only achievable in the limit of infinite spatial resolution, but even such an approximation is nontrivial to obtain in reality because the geometric primitives overlap. As a consequence, no closed‐form solution exists for computing the radii corresponding to a desired volume. To calculate voxel sizes, the validation procedure uses the same field of view (FOV) provided to the phantom‐generation function (default: 30×30×30 cm). It divides the FOV by the number of voxels along each axis to determine the dimensions of a single voxel, from which it calculates the voxel volume. Then it counts the voxels with an intensity value matching the expected lung or heart chamber intensity, and determines the organ's volume as the product of the number of voxels and the volume of a single voxel. Finally, the relative error is computed as the ratio of the measured to the expected volume values.

**FIGURE 4:**
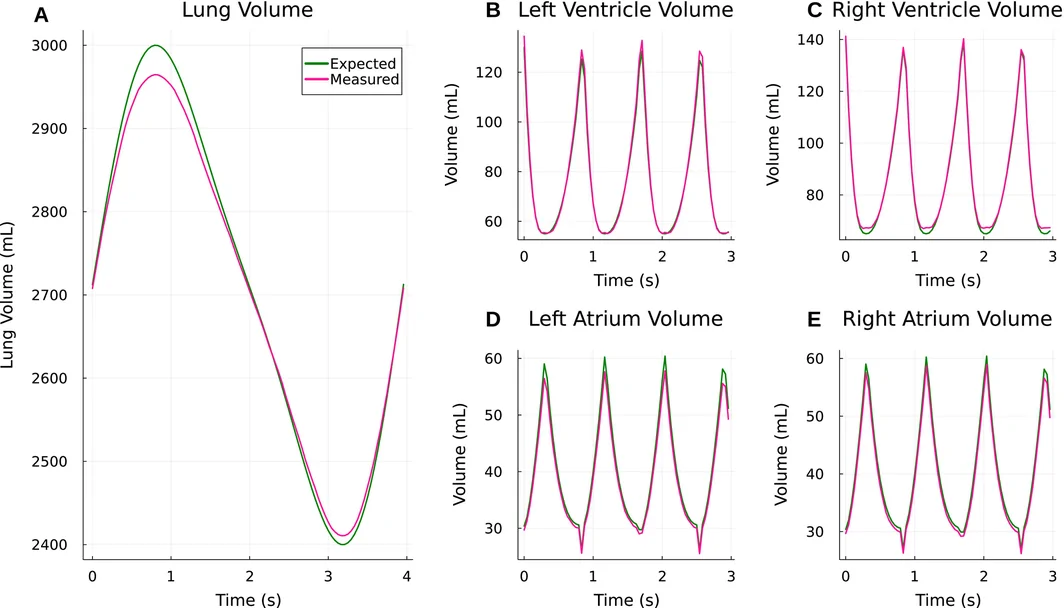
Validation results for a 4D phantom dataset generated using the signals from built‐in respiratory and cardiac signal generators. Each plot compares the expected (green) and measured (magenta) volume trajectories. Subplot A shows expected and measured lung volumes, and B–E subplots display these volumes for the four cardiac chambers: (B) left ventricle, (C) right ventricle, (D) left atrium, and (E) right atrium.

For the respiratory signal, the maximum relative error was 5.5%, and the median error was 2.5% across the full supported range of lung volumes (1.2–6.0 L). Within the range of normal resting‐state lung volumes (2.4–3.0 L), the phantom followed the input signal more closely, with a maximal relative error of 1.2% and a median error of 0.5%. The cardiac‐chamber volumes also showed low errors, with maximal relative error of 3.64%, 1.76%, 3.67%, and 2.57% for the left ventricle, right ventricle, left atrium, and right atrium, respectively. Figure 4 shows the difference between the expected and the measured volumes, using the schematic respiratory and cardiac signals from the built‐in signal generator.

Experiment C evaluates the computational performance of the reference implementation. The script written for this experiment generates multiple data sets in varying sizes, with different numbers of time frames and computing threads. Benchmarking results are summarized in Table 1. The measurements were conducted on a Linux‐based server with AMD EPYC 7352 processors, with Julia v1.12.5 and GeometricMedicalPhantoms.jl v1.0.3. The running times were measured using BenchmarkTools.jl, the de facto standard for measuring Julia code execution times. Table 1 summarizes the minimum running times for phantom generation across various parameter combinations.

**TABLE 1:** Benchmarking results of the reference implementation with varying sizes, time frames, and thread counts.

Dim.	Size	Frames	Threads	Runtime
2D	$128^{2}$	1	1	166.871 μs
		100	1	12.809 ms
			2	6.846 ms
			4	3.589 ms
			8	3.044 ms
	$256^{2}$	1	1	438.89 μs
		100	1	30.277 ms
			2	16.936 ms
			4	12.348 ms
			8	4.681 ms
3D	$128^{3}$	1	1	13.219 ms
		100	1	853.69 ms
			2	418.298 ms
			4	237.191 ms
			8	163.744 ms
	$256^{3}$	1	1	116.159 ms
		100	1	9.114 s
			2	4.576 s
			4	2.55 s
			8	1.776 s

Finally, Experiment D demonstrates a realistic Cartesian MRI simulation. Although Bloch simulation is required for the most realistic results, a much simpler method is sufficient to demonstrate motion artifacts. Our approach assumed the following: (1) the discrete Fourier transform gives a good approximation of k‐space coefficients, (2) motion during the readout event in a single line of k‐space is negligible, but (3) motion needs to be taken into account during phase encoding (between frequency encoding lines). In this experiment, we constructed a simulated k‐space line‐by‐line, with motion simulated between lines, performed a basic reconstruction, and then visualized the difference between the reconstructed image and a single frame of the phantom from the middle of the simulated time range.

**FIGURE 5:**
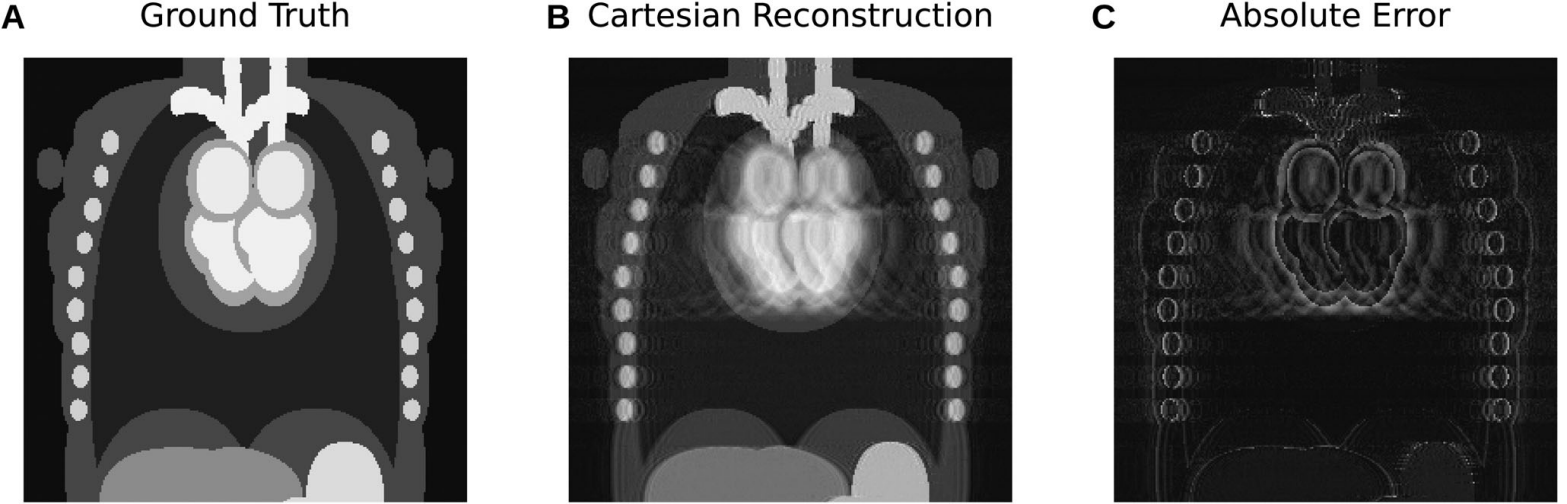
Result of Experiment D, which simulates a conventional Cartesian MRI acquisition and demonstrates the motion artifacts after a basic reconstruction. Image A shows the central coronal slice from a frame near the middle of the simulated time span; the reconstruction result is shown in image B; and image C shows the difference between the left and the middle images.

First, a dynamic 2D phantom with respiratory and cardiac motion was generated at a temporal resolution of 50 ms over the simulated time range of 12.8 s. The heart rate was 72 BPM, while the respiratory rate was 12 BPM. The spatial Fourier transform was then applied to each image. When constructing the simulated “raw data,” this simulated acquisition mimics the line‐by‐line nature of phase‐encoding in real MRI: k‐space was built by extracting a single phase‐encoding line from each successive transform, corresponding to how an MRI scanner would sample one line of k‐space per radio‐frequency pulse with 50 ms repetition time. The resulting data was reconstructed via an inverse Fourier transform and was compared with the ground‐truth phantom to quantify the motion‐induced blurring and artifacts. Figure 5 shows the ground truth, the reconstructed image, and their difference.

## 4 Discussion

The presented phantom introduces an intermediate layer between standard static phantoms, such as the Shepp–Logan phantom, and highly realistic 4D in silico and in vitro solutions, which are often overly complex for a broad range of research tasks. It provides an easy‐to‐use tool for researchers developing new reconstruction algorithms and toolboxes, enabling rapid testing and clear demonstration of methodological advances. The primary strength of the phantom lies in its high degree of customization, including parametrization of spatial and temporal resolution, cardiac and pulmonary volume trajectories, and organ‐specific intensity values. To facilitate integration with the phantom into reconstruction pipelines, an open‐source, high‐performance reference implementation is provided as both a Julia library and a standalone console application.

The capabilities of the phantom and its reference implementation were demonstrated through four experiments. The first experiment illustrated the dynamics of the phantom, showing how the values along a horizontal and a vertical line change over time due to respiratory and cardiac motion. The second experiment quantified the difference between the input volumes and the volumes computed from voxel count for the lung and the four cardiac chambers, demonstrating low relative errors across a range of volumes. The third experiment evaluated the computational performance of the reference implementation, showing that it can generate large 4D datasets in a reasonable time frame, especially when utilizing multiple threads. Finally, a simplistic Cartesian MRI simulation demonstrated how the phantom can be used to quantify motion blur and artifacts in reconstructed images.

This work does not aim to replace the existing anatomically and physiologically realistic phantoms, nor does it diminish the need for in vivo validation. While the phantom models some aspects of the complexity of dynamic imaging, it vastly simplifies the shapes of the human anatomical morphology and lacks texture. Consequently, it is not suitable for fine‐tuning acquisition or reconstruction parameters, nor for validating reconstruction and segmentation algorithms.

## 5 Conclusion

In conclusion, the proposed dynamic geometric phantom provides an intermediate solution between simple static phantoms and complex, proprietary, anatomically realistic 4D models. It offers an easy‐to‐use tool for researchers developing new reconstruction algorithms and toolboxes, enabling rapid testing and clear demonstration of their methods. Its core strengths lie in its high degree of customization, enabling parameterization of spatial and temporal resolution, heart chamber and lung volumes, and specific organ intensity values. An open‐source, high‐performance reference implementation in Julia is also presented, available as both a library and a standalone console application, making it easy to integrate the phantom into various reconstruction pipelines.

## Funding

This work was supported by the Hungarian National Research, Development and Innovation Office through the Cooperative Doctoral Programme for Doctoral Scholarships grant EKÖP‐KDP 2024, the European Union within the framework of the Artificial Intelligence National Laboratory grant MILAB, RRF‐2.3.1‐21‐2022‐00004, and by the European Union's Horizon 2020 Research and Innovation Program, grant agreement no. 739593: HCEMM, supported by the EU Program H2020‐EU.4.a, and by Research Grant Hungary 151414 from the National Research, Development and Innovation Office, Government of Hungary.

## Data Availability

The source code for the reference implementation of the presented phantom is available on GitHub at https://github.com/hakkelt/GeometricMedicalPhantoms.jl and is registered under the MIT license as a Julia package named GeometricMedicalPhantoms.jl. The current version of the package is 1.0.3, with the following DOI provided by the Zenodo platform: 10.5281/zenodo.20259159. The C, Python and MATLAB wrappers and the standalone application can be downloaded at https://github.com/hakkelt/GeometricMedicalPhantoms.jl/releases/tag/v1.0.3. The software documentation is available at https://hakkelt.github.io/GeometricMedicalPhantoms.jl/v1.0.3/. The source code and the results of experiments described in the results section are published at https://github.com/hakkelt/TorsoPhantomDemo. The reference implementation is considered feature‐complete, and we will provide bug‐fix support for at least 10 years. The phantom was implemented as part of an industrial program, and therefore, the support might be extended.
